# *APOA2* Polymorphism in Relation to Obesity and Lipid Metabolism

**DOI:** 10.1155/2013/289481

**Published:** 2013-12-09

**Authors:** Moushira Erfan Zaki, Khalda Sayed Amr, Mohamed Abdel-Hamid

**Affiliations:** ^1^Medical Research Division, Biological Anthropology Department, National Research Centre, Cairo 12622, Egypt; ^2^Human Genetics and Genome Research Division, Medical Molecular Genetics Department, National Research Centre, Cairo 12622, Egypt

## Abstract

*Objectives*. This study aims to analysis the relationship between c.-492T>C polymorphism in *APOA2 *gene and the risk for obesity in a sample of Egyptian adolescents and investigates its effect on body fat distribution and lipid metabolism. *Material and Methods*. A descriptive, cross-sectional study was conducted on 303 adolescents. They were 196 obese and 107 nonobese, aged 16–19 years old. Variables examined included body mass index (BMI), waist circumference (WC), waist to hip ratio (WHR), systolic and diastolic blood pressure (BP), body fat percentage (BF%), abdominal visceral fat layer, and dietary intake. Abdominal visceral fat thickness was determined by ultrasonography. The polymorphism in the *APOA2* c.-492T>C was analyzed by PCR amplification. *Results*. Genotype frequencies were in Hardy-Weinberg equilibrium. The frequency of the mutant C allele was significantly higher in obese cases compared to nonobese. After multivariate adjustment, waist, BF% and visceral adipose layer, food consumption, and HDL-C were significantly higher in homozygous allele CC carriers than TT+TC carriers. *Conclusions*. Homozygous individuals for the C allele had higher obesity risk than carriers of the T allele and had elevated levels of visceral adipose tissue and serum HDL-C. Moreover, the study shows association between the *APOA2* c.-492T>C polymorphism and food consumption.

## 1. Introduction

Obesity-linked genetic variations in the presence of other routine habits such as smoking, physical inactivity, and unhealthy food intake may greatly raise the risk of a person developing heart diseases (cardiovascular diseases, CVD).

Excess body fat, obesity, is one of the most common disorders in clinical practice. The location of the body fat is a major determinant of the degree of excess morbidity and mortality due to obesity [1]. At least two components of body fat are associated with obesity-related adverse health outcomes. These are the amount of subcutaneous truncal or abdominal fat, and the amount of visceral fat located in the abdominal cavity. Each of these components of body fat is associated with varying degrees of metabolic abnormalities and independently predicts adverse health outcomes. Many complex traits are thought to be inherited since they often run in families. However, these complex traits do not show typical mendelian pedigree patterns. These nonmendelian diseases may depend on several susceptibility loci, with a variable contribution from environmental factors. Discovering the major susceptibility locus may be the key to advances in understanding the pathophysiology of a disease.

There have been several studies using association approaches in order to undertake systematic searches for candidate genes in obesity defined as elevated body mass index (BMI, kg/m^2^) [2].

Apolipoprotein A-II (APOA-II) is the second most common protein in high-density lipoproteins. APOA-II appears to impair the reverse cholesterol transport and antioxidant function of high-density lipoprotein, which is consistent with the observation that increased APOA-II levels promote the development of atherosclerosis [3]. *APOA2* polymorphism (-265T>C) has been renamed to c.-492T>C, according to the Human Genomic Variation Society version 2012. A functional polymorphism representing a T-to-C substitution at the -492 position of this gene has been associated with waist circumference and lower levels of plasma APOA-II in European men [4], suggesting that genetic variation at the *APOA2* may be associated with body fat distribution phenotypes. Lower levels of visceral adipose tissue (VAT), both absolute and relative to their total body fat, have been reported in African-American compared with white women [5, 6], which may be related to differences in genetic make-up between women of different ethnic backgrounds.

New obesity loci continue to be identified through genome-wide association studies in populations of increasing size and ethnic diversity [7, 8] but understanding of the mechanisms by which known genetic variants contribute to obesity remains limited. Several well established obesity candidates encode proteins that appear to modulate obesity risk via energy intake, a key determinant of obesity risk [9].

In a previous investigation carried out on White Americans participating in the Genetics of Lipid Lowering Drugs and Diet Network (GOLDN) study, the recessive effects for the c.-492T>C polymorphism was observed [10]. Homozygous individuals for the C allele had higher body mass index (BMI) and obesity risk than the carriers of the T allele, but relationships between *APOA2* c.-492T>C genotype and obesity among Egyptian adolescents are unexplored till now.

Therefore, our objectives were to analyzing the association between the *APOA2 *c.-492T>C polymorphism SNP and the risk of obesity and study its association with anthropometric measurements, body fat distribution, food consumption, and lipid metabolism in a sample of Egyptian adolescents.

## 2. Materials and Methods

A descriptive cross-sectional study was conducted on randomly selected 303 Egyptian adolescents. They were 196 obese and 107 nonobese. Their age ranged 16–19 years old and the mean age was 17.45 ± 2.54 years. Obese cases had BMI greater than 95th percentile for age and gender according to the National Egyptian Growth Curves of Children and Adolescents [11].

The data were collected from June 2011 to July 2012 and were extracted from a project entitled “Obesity among Youth: Lifestyle and Genetic Factors” funded by the Science and Technology Development Fund (STDF), Egypt. This study protocol was approved by the ethical committee board of the National Research Centre of Egypt (no. 10/223). An informed written consent was obtained from all participants. All individuals were clinically evaluated and anthropometric data were collected.

### 2.1. Anthropometric Measurements

Anthropometric variables including height, weight, waist, and hip were measured. Body weight was measured with the patients in light clothing and without shoes. Height was measured with the patients standing with their backs leaning against the stadiometer of the same scale. BMI was calculated as weight in kilograms divided by height in meters squared (kg/m^2^). WC and hip circumference (HC) were measured in cm using a plastic, nonstretchable tailor's tape. WC was measured with light clothing at a level midway between the lower rib margin and the iliac crest standing and breathing normally. HC was measured at the level at the widest circumference over the buttocks (at the greater trochanter). Subsequently, the waist hip ratio (WHR) was calculated as WC divided by HC. Anthropometric measurements were obtained according to standardized equipment and following the recommendations of the International Biological Program [12].

Systolic and diastolic blood pressure (BP) were measured with the patients sitting with their left arm at heart level using a professional Riester sphygmomanometer manufactured in Japan. Several measurements were made, from which an average BP measurement was obtained. BF% was measured by Tanita Body Composition Analyzer (SC-330).

### 2.2. Genotyping

Genomic DNA was extracted from peripheral blood leukocytes using GeneJET Genomic DNA Purification Kit—(Fermentas, German) according to the manufacturer instructions. Genotyping of c.-492T>C polymorphism in the *APOA2* gene was carried out using polymerase chain reaction-restriction fragment length polymorphism (PCR-RFLP) analysis [4]. Two pair of primers were used to amplify the promoter region of the *APOA2* gene containing the polymorphism; upstream primer 5′CAT GGG TTG ATA TGT CAG AGC-3′ and downstream primer 5′ TCA GGT GAC AGG GAC TAT GG 3′.

PCR was carried out in a 25 *μ*L total final volume containing 200 *μ*M dNTPs (Finzyme, Finland), 10 pmole of each primer, 2 U of Taq polymerase (Finzyme, Finland), and 500 ng DNA. Thermal cycling conditions were as follows: denaturation at 95°C for 10 min, followed by 30 cycles of denaturation at 95°C for 30 sec, annealing at 59.5°C for 30 sec, and elongation at 72°C for 30 sec followed by a final elongation of 5 min.

Ten *μ*L of successfully amplified PCR products were digested with Fast Digest BsmI enzyme (Fermentas, Germany), incubated at 37°C for 5 min and the fragments were run in 3% agarose gel stained with ethidium bromide, and analyzed under ultraviolet light. The BsmI enzyme cuts the PCR product (273 bp) in two fragments 215 and 58 bp in presence of the T allele.

### 2.3. Abdominal Ultrasonographic Examination

Ultrasonography was carried out by using GE logic *α* 200-ultrasound machine. Visceral fat layer was measured from the region just above the umbilicus [13]. The convex-array probe (3.5 MHz) was used for measuring visceral abdominal fat and anterior wall of the aorta.

### 2.4. Dietary Intake

Food intake carried out using 24 hours dietary recall. Cases were asked to recall their dietary intakes of the previous 24 hours. In particular, we asked about intake of carbonated beverages, juices, and other casual intake. Food frequency method assessed food consumption frequencies per day and week and month basis by using a questionnaire. It was focused on different kinds of food consumption frequencies rather than consumption of specific nutrients. The energy and nutrient contents were computed.

### 2.5. Biochemical Analyses

HDL cholesterol was measured after precipitation of non-HDL cholesterol with magnesium/dextran. We measured LDL cholesterol by use of a homogeneous direct method (LDL Direct Liquid Select Cholesterol Reagent; Equal Diagnostics) on the Hitachi autoanalyzer 704 (Roche Diagnostics Switzerland).

### 2.6. Statistical Analysis

Quantitative variables were expressed as mean ± S.D., and qualitative variables were expressed as percentages. Differences between groups were tested using an independent two-sample *t*-test and chi-square test was used to test for differences in the distribution of categorical variables. *P* values < 0.05 were considered statistically significant.

## 3. Results

The characteristics of the obese cases and nonobese are given in Table 1, where we compare the anthropometric and clinical variables for the obese and nonobese individuals. There were significant differences in BMI, WC, WHR, BF%, and visceral fat thickness between the two groups. Obese adolescents had higher values than nonobese in both genders. *APOA2 *genotype frequencies did not deviate from Hardy-Weinberg equilibrium expectations and did not differ between males and females. Therefore, males and females were analyzed together. The genotype and allele distribution are presented in Table 2. The data indicate that genotype tends to differ significantly between obese and nonobese adolescents in the CC and TT genotypes, remaining significant when the genotypes in CC and CT+TT were grouped; the CC genotype was more common in obese cases than in nonobese (CC frequency 32.1% in obese cases and 9.3% in lean controls, *P* < 0.001). The allele frequency of the *APOA2* c.-492T>C polymorphism was also significantly different between the two groups (Table 2).

*APOA2* genotype was evaluated by comparing homozygous minor allele carriers (CC) with combined homozygous major (TT) and heterozygous (TC) subjects (Table 3). After adjusting for age, sex, and BMI, anthropometric measures showed significant differences between homozygous and heterozygous carriers. BMI, WC, BF%, and visceral fat were significantly higher in CC subjects compared with combined heterozygotes (TC) and homozygous major (TT) carriers (*P* < 0.001). Significant elevated HDL-C was observed in CC subjects compared with the carriers of T allele.

Homozygous individuals for the CC allele had a statistically higher mean of energy intake, total fat intake (g/day), and saturated fat (SATFAT) than carriers of the T allele.

## 4. Discussion

The present study found strong association between the *APOA2* c.-492T>C SNP polymorphism and obesity risk and anthropometric measures. The study observed that CC homozygotes had higher BMI, WC, BF%, visceral fat, food consumption, and HDL-C than carriers of the T allele. These results were consistent with the findings of the previous study of overweight individuals in other populations. Relatively few studies reported the association between *APOA2* polymorphisms and phenotypic traits [14–17]. Few genetic variants have been identified in the *APOA2* gene [18]. Interestingly, a T-C transition at position -492 affecting element of the *APOA2* promoter has been reported to be functional in 2 independent studies, both demonstrating an −30% drop in basal transcription activity [4, 19]. In one of these studies, the *APOA2* polymorphism was associated with waist circumference in men [4]. Another study [19] reported an association between this polymorphism and abdominal fat depots in women.

Association between *APOA2* c.-492T>C SNP and BMI or obesity only in the presence of high-saturated fat intake in three American populations has been observed [20]. Moreover, with this gene-diet interaction other studies extend the findings to other geographical areas (Europe and Asia), reporting that when saturated fat intake is low (<22 g/d), this SNP does not have any effect on BMI or obesity. However, when saturated fat intake is high (≥22 g/d), significant differences in anthropometric variables were detected between CC individuals and T allele carriers. Further adjustment for other macronutrients did not change the significance of these findings, supporting the specificity of saturated fat as a driver of this interaction [21]. Moreover, other study reported genotype-associated differences in specific intake-related behaviours, which may contribute to obesity risk, identifying the possible role of ghrelin in modulating *APOA2*-nutrient interactions [22]. Eating behaviours have been identified as related to obesity risk [23, 24] and appear to be associated with *APOA2* genotype in a manner consistent with obesity risk. Relationship between *APOA2*, saturated fat, and hormonal regulation of food intake has also been identified, which may be relevant to weight control. The interactions between *APOA2* and saturated fat for obesity may be mediated via modulation of plasma ghrelin and expansion of knowledge of APOA2 and obesity to include modulation of specific behaviours and hormonal mediators not only broadens understanding of gene-diet interactions, but also facilitates the pragmatic, future goal of developing dietary guidelines based on genotype [22]. Lower saturated fat was associated with lower ghrelin in CC carriers, which may theoretically be expected to accompany lower energy intake and smaller body size.

Despite the scarcity of previous data supporting a role of APOA2 in regulating food intake, numerous experimental evidence demonstrates a pivotal role of another apolipoprotein, APOA4, as a satiety signal [25, 26]. Fujimoto et al. [25] were the first to report that APOA4 is a satiety factor secreted by the intestine after fat absorption and that this function of APOA4 is not shared by gut APOA1. APOA2 is a member of the apolipoprotein multigene super family, which includes genes encoding soluble apolipoproteins (e.g., APOA1 and APOA4) that share genomic structure and several functions. Although all these apolipoprotein genes have been found to be related to obesity in at least one epidemiological study [27], only APOA4 has been subscribed in regulation of food intake, acting as a satiety signal. The present study shows association between the *APOA2* polymorphism and food consumption, suggesting a potential new role of APOA2 in the regulation of human appetite. Moreover, the present study shows that the c.-492T>C locus is an important genetic determinant of HDL cholesterol concentration. The mechanisms of this proatherogenic capability of increased human apoA-II could be due to increased concentration of apoB-containing lipoprotein and decreased HDL cholesterol, impairment of reverse cholesterol transport due to decreased cholesterol efflux and esterification [28, 29].

In summary, the present study emphasized that the homozygous individuals for the CC allele had higher obesity risk than carriers of the T allele. The functional polymorphism representing a T-to-C substitution at the -492 position of this gene is associated with visceral adipose tissue and food consumption. Moreover, this polymorphism had a significant role on HDL cholesterol concentration and could be a modifier gene for familial combined hyperlipidemia.

## Figures and Tables

**Table 1:** General characteristics of obese and non-obese adolescents.

Parameters	Females		Males	
	Obese	Non-obese	Obese	Non-obese
BMI (kg/m^2^)	34.40 ± 5.96**	21.99 ± 3.45	32.75 ± 6.075**	19.86 ± 3.33
Waist (cm)	95.90 ± 12.23**	72.57 ± 8.94	98.05 ± 18.90**	71.79 ± 8.54
WHR	0.82 ± 0.06**	0.77 ± 0.16	0.88 ± 0.129**	0.81 ± 0.06
BF%	33.74 ± 13.05**	30.96 ± 12.93	33.88 ± 13.78**	26.63 ± 11.61
Systolic BP (mmHg)	110.77 ± 14.81	109.60 ± 13.09	110.67 ± 16.37	108.80 ± 13.23
Diastolic BP (mmHg)	71.79 ± 10.31	71.80 ± 7.93	71.42 ± 9.746	73.00 ± 10.00

**Statistically significant differences (*P* < 0.01) between obese and non-obese subjects.

**Table 2:** Genotype and allelic distribution of *APOA2* c.-492T>C polymorphisms.

Polymorphisms	Genotypes			Alleles		Grouped genotypes	
	CC	TC	TT	C	T	CC	TT+TC
Controls (*n* = 107)	10	64	33	84	130	10	97
	9.34%	59.81%	30.84%	39.25%	60.74%	9.34%	90.65%
Obese (*n* = 196)	63	62	71	188	204	63	133
	32.1%	31.6%	36.2%	47.96%	52.04%	32.1%	67.9%
*P* value	0.001	0.001	0.022	0.01	0.01	0.001	0.001

**Table 3:** Associations of *APOA2* c.-492T>C polymorphism genotype with anthropometric parameters^a^.

Parameters	CC (*n* = 73)	TT+TC (*n* = 230)
BMI (kg/m^2^)	32.29 ± 7.48*	23.77 ± 8.25
Waist (cm)	87.91 ± 15.81*	82.84 ± 17.04
WHR	0.89 ± 0.11	0.82 ± 0.19
BF%	35.69 ± 11.37*	30.87 ± 12.43
Systolic BP (mmHg)	108.70 ± 16.24	110.66 ± 13.89
Diastolic BP (mmHg)	71.30 ± 10.41	72.11 ± 9.08
Energy intake (Kcal/d)	1957.1 ± 87.9*	1499.7 ± 87.1
Total fat (g/d)	93.6 ± 31.6*	51.5 ± 29.5
SATFAT (g/d)	33.9 ± 8.95*	22.2 ± 7.91
HDL-C (mg/dL)	56.60 ± 7.88*	38.41 ± 7.81
LDL-C (mg/dL)	115.68 ± 38.87	110.64 ± 36.10

*Statistically significant differences (*P* < 0.05) between CC homozygous subjects and carriers of the T allele for the corresponding variable.

^a^Data are adjusted for age, gender, and BMI.
